# Bacterial lipopolysaccharides form procollagen-endotoxin complexes that trigger cartilage inflammation and degeneration: implications for the development of rheumatoid arthritis

**DOI:** 10.1186/ar4291

**Published:** 2013-09-10

**Authors:** Wolfgang Lorenz, Constanze Buhrmann, Ali Mobasheri, Cora Lueders, Mehdi Shakibaei

**Affiliations:** 1 Institute of Indoor Diagnostics, Marconistrasse 23, D-40589 Duesseldorf, Germany; 2 Musculoskeletal Research Group, Institute of Anatomy, Ludwig Maximilian University Munich, Pettenkoferstrasse 11, D-80336 Munich, Germany; 3 Medical Research Council-Arthritis Research UK Centre for Musculoskeletal Ageing Research, Arthritis Research UK Pain Centre, Arthritis Research UK Centre for Sport, Exercise, and Osteoarthritis, Faculty of Medicine and Health Sciences, The University of Nottingham, Sutton Bonington Campus, Sutton Bonington, LE12 5RD, Nottingham, United Kingdom; 4 Center of Excellence in Genomic Medicine Research (CEGMR), King AbdulAziz University, Jeddah 21589, Kingdom of Saudi Arabia; 5 Schools of Pharmacy and Life Sciences, University of Bradford, Richmond Road, Bradford, BD7 1DP, United Kingdom; 6 German Heart Institute Berlin, Department of Thoracic and Cardiovascular Surgery, Laboratory for Tissue Engineering, Augustenburger Platz 1, 13353 Berlin, Germany

## Abstract

**Introduction:**

We have previously reported that bacterial toxins, especially endotoxins such as lipopolysaccharides (LPS), might be important causative agents in the pathogenesis of rheumatoid arthritis (RA) in an *in vitro *model that simulates the potential effects of residing in damp buildings. Since numerous inflammatory processes are linked with the nuclear factor-κB (NF-κB), we investigated in detail the effects of LPS on the NF-κB pathway and the postulated formation of procollagen-endotoxin complexes.

**Methods:**

An *in vitro *model of human chondrocytes was used to investigate LPS-mediated inflammatory signaling.

**Results:**

Immunoelectron microscopy revealed that LPS physically interact with collagen type II in the extracellular matrix (ECM) and anti-collagen type II significantly reduced this interaction. BMS-345541 (a specific inhibitor of IκB kinase (IKK)) or wortmannin (a specific inhibitor of phosphatidylinositol 3-kinase (PI-3K)) inhibited the LPS-induced degradation of the ECM and apoptosis in chondrocytes. This effect was completely inhibited by combining BMS-345541 and wortmannin. Furthermore, BMS-345541 and/or wortmannin suppressed the LPS-induced upregulation of catabolic enzymes that mediate ECM degradation (matrix metalloproteinases-9, -13), cyclooxygenase-2 and apoptosis (activated caspase-3). These proteins are regulated by NF-κB, suggesting that the NF-κB and PI-3K pathways are involved in LPS-induced cartilage degradation. The induction of NF-κB correlated with activation of IκBα kinase, IκBα phosphorylation, IκBα degradation, p65 phosphorylation and p65 nuclear translocation. Further upstream, LPS induced the expression of Toll-like receptor 4 (TLR4) and bound with TLR4, indicating that LPS acts through TLR4.

**Conclusion:**

These results suggest that molecular associations between LPS/TLR4/collagen type II in chondrocytes upregulate the NF-κB and PI-3K signaling pathways and activate proinflammatory activity.

## Introduction

Rheumatoid arthritis (RA) is a systemic and chronic inflammatory disease that occurs in 0.5 to 1.0% of the adult population worldwide [1]. It is characterized by hyperplasia of the synovial lining cells, increase in macrophages, high levels of proinflammatory cytokines, such as IL-1β and TNF-α, expression of autoantibodies and upregulation of catabolic matrix degrading enzymes such as matrix metalloproteinases (MMPs), and serine proteases leading to progressive destruction of cartilage and bone [2-4]. RA can lead to joint and cartilage damage, significant disability, and reduction in quality of life. RA is a multifactorial disease and classified as an autoimmune disorder, that primarily affects the small diarthrodial joints of the hands and feet and affects multiple joints throughout the body [5]. Although the etiology of RA is not yet fully understood [6], it is believed to be caused by a combination of environmental (microbial and viral triggers), immunomodulatory, genetic predisposition factors and a number of inflammatory pathways in response to endogenous and/or exogenous antigens [7]. These factors play essential roles in the pathogenesis of RA.

A prominent feature of RA is the T-cell infiltrates that suggest these cells are key participants in RA [8,9]. Moreover, macrophage-like and fibroblast-like synoviocytes proliferate and form a pannus, which destroys cartilage and subchondral bone leading to loss of joint function [10]. Activated macrophages and synoviocytes produce soluble mediators and proinflammatory cytokines including TNF-α and IL-1β, which play a major role during RA, directing upregulation of other proinflammatory cytokines, increasing synovial cellular infiltration, macrophages, osteoclast and chondrocyte activation and increasing angiogenesis [11,12].

It is known that lipopolysaccharides (LPS) are the main endotoxin components of gram-negative bacterial cell walls. They activate immune cells, such as macrophages and neutrophils in the host and in turn, the stimulated cells synthesize proinflammatory factors, such as IL-1β and TNF-α, matrix proteases and free radicals and thus lead to dramatic secondary inflammation in tissues [13,14]. Further, LPS is used to establish transient synovitis-osteoarthritis models for therapeutic research [15]. LPS-induced signaling is thought to begin with its binding to specific surface receptors such as Toll-like receptor 4 (TLR4), which trigger intracellular signaling cascades leading to activation of the multiple proinflammatory signaling pathways [16,17]. Moreover, LPS is the primary ligand of TLR4, activating it through binding to its accessory protein MD-2 [18].

It has been previously suggested that the inhabitants of buildings with microbiological infestation caused by dampness through, for example, water damage have an increased risk of RA [19-21]. We also observed a connection between microbial infestation of buildings after water damage and RA manifestation in inhabitants [19], where symptoms of RA decreased in patients after removing damp walls, with 26% of patients completely recovered [19]. In a previous *in vitro *study, we have demonstrated that in primary isolated chondrocytes, bacterial endotoxins respectively LPS from damp walls in buildings, dose-dependently increased MMP-3 production and dramatically suppressed collagen type II production [19].

Several lines of evidence suggested that proinflammatory cytokines and LPS stimulate multiple signaling pathways such as the phosphatidylinositol 3-kinase (PI-3K)/protein kinase B (Akt), mitogen-activated protein kinase (MAPK) and nuclear factor-κB (NF-κB) [22,23]. Several reports have suggested that PI-3Ks are involved in the cytokine signaling pathways and inflammatory processes and mediate activation and translocation of NF-κB through targeting IκB kinase (IKK)-α kinase or phosphorylation of p65, a process that is inhibited by the PI-3K-specific inhibitor wortmannin [24,25]. PI-3K activates Akt one of the main downstream kinases in different cells [26]. Furthermore, NF-κB is activated in the synovium in humans and animals, supporting an essential role for this transcription factor in cartilage destruction in RA [27,28]. The inhibited subunits of NF-κB are trapped in the cytoplasm as a complex by association with an inhibiting IκBα subunit. Through the phosphorylation, IκBα dissociates from the complex and the p65 and p50 subunits freely translocate to the cell nucleus and bind to NF-κB recognition sites in the promoter regions of various NF-κB-regulated genes. Activated NF-κB is known to be involved in the regulation of a wide array of genes; among them are those in the infection, adhesion, cell cycle, apoptosis, survival and inflammatory process by upregulating the transcriptional levels of multiple genes, including IL-1β, TNF-α, IL-6, cyclooxygenase-2 (COX-2) and matrix metalloproteinases (MMPs) [29-31]. NF-κB appears to be a common target of multiple converging catabolic signaling pathways mediated by proinflammatory cytokines.

Hence, in this study we have investigated in an *in vitro *model of human chondrocytes: (1) the presence of LPS in the extracellular matrix (ECM) in cartilage and its binding potential to collagen fibrils (procollagen-endotoxin complex, (PEC)), (2) whether LPS upregulate the inflammatory effects, which are responsible for matrix degradation, inflammation and apoptosis and (3) whether LPS/TLR4 association, at least in part, activates NF-κB and PI-3K/Akt signaling pathways.

## Materials and methods

### Antibodies

Antibodies against collagen type II (AB746), alkaline phosphatase-linked sheep anti-mouse and sheep anti-rabbit secondary antibodies for immunoblotting were purchased from Millipore (Schwalbach, Germany). Polyclonal anti-active caspase-3 and monoclonal MMP-9 and -13 antibodies recognizing both proenzyme and activated enzyme were obtained from R&D Systems (Abingdon, UK). Monoclonal anti-β-actin and normal rabbit IgG were purchased from Sigma-Aldrich Chemie (Munich, Germany). Antibodies against phospho-specific IκBα (Ser 32/36) and against anti-phospho-specific p65 (Ser536) were obtained from Cell Technology (Beverly, MA, USA). Anti-IκBα kinase (IKK)-α and anti-IKK-β were obtained from Imgenex (Hamburg, Germany). Monoclonal (anti-poly (ADP-ribose) polymerase) (PARP) antibodies were purchased from Becton Dickinson (Heidelberg, Germany). Cyclooxygenase-2 antibody was obtained from Cayman Chemical (Ann Arbor, MI, USA). Polyclonal antibody against TLR4 was obtained from Santa Cruz Biotechnology (Santa Cruz, CA, USA). Secondary antibodies for immunofluorescence were purchased from Dianova (Hamburg, Germany). All antibodies were used at concentrations and dilutions recommended by the manufacturer. Monoclonal anti-*Escherichia coli *LPS antibody (2D7/1) was obtained from Abcam (Cambridge, UK). Polyclonal rabbit anti-*E.coli *O6 serum was kindly provided by Prof. P. Roggentin (Institute für Hygiene und Umwelt, Hamburg, Germany), against *E.coli *Nissle 1917 (DSM 6601, serotype O6:K5:H1).

### Growth media, chemicals and endotoxin

Growth medium (Ham's F-12/Dulbecco's modified Eagle's medium (DMEM) (50/50) containing 10% fetal calf serum (FCS), 25 mg/ml ascorbic acid, 50 IU/ml streptomycin, 50 IU/ml penicillin, 2.5 mg/ml amphotericin B, essential amino acids, and L-glutamine) was obtained from Seromed (Munich, Germany). BMS-345541 and Trypsin/EDTA (EC 3.4.21.4) were purchased from Sigma-Aldrich. Epon and LR-white were obtained from Plano (Marburg, Germany). Wortmannin was purchased from Biomol (Plymouth Meeting, PA, USA). LPS from *E.coli *endotoxins (E8029) was purchased from Sigma-Aldrich and dissolved in phosphate-buffered saline (PBS) at 1 mg/ml.

### Experimental design

Based on our observations during the last years, we have formulated the hypothesis that bacteria or bacterial metabolites may be important causative agents for rheumatic diseases [19]. However, it is not possible to identify all bacteria in the samples from damp buildings, as some species will not grow on agar. We further observed that exercised and constantly used joint areas are specially affected. Therefore, we assumed that very fine particles, molecule clusters or molecular aggregates that are inhaled from the inhabitants would partly be transported to the joints or the cartilage through circulation. We further hypothesized that endotoxins may interfere directly with the mechanism for the synthesis and assembly of collagen fibres.

We performed experiments in this study on primary human chondrocytes (HCHON) purchased from Provitro (Berlin, Germany), to mimic cellular events that occur in the clinical condition of osteoarthritis (OA) or RA. To stimulate inflammatory processes in chondrocytes, we adopted a model that stimulates cells with LPS (0 to 1000 ng/ml). During monolayer expansion chondrocytes were cultured in whole-cell culture medium containing 10% FCS. Chondrocytes were washed three times with serum-starved medium (containing only 0.5% FCS) and further incubated for 30 min with the same medium before initiating treatment with LPS and/or inhibitors.

In a second approach, chondrocytes in monolayer cultures, treated as above, were transferred to high-density cultures and cultured under identical conditions with serum-starved medium to examine the effects of LPS and/or inhibitors on chondrocyte differentiation potential in a three-dimensional environment. Three-dimensional high-density culture was performed as previously described [32]. Briefly, primary cultures of human chondrocytes (1 × 10^6^) were pipetted onto a nitrocellulose filter (pore diameter 0.2 µm, Sartorius, Göttingen, Germany) resting on a steel net bridge. Culture medium reached the filter medium interface and cells were nurtured through diffusion. After one day in culture, cells aggregated and formed a pellet on the filter. Cultures were grown at 37°C in a humidified atmosphere with 5% CO_2_.

For investigation of NF-κB translocation and IκBα phosphorylation, human chondrocyte cultures were treated either with LPS or co-treated with LPS and/or inhibitors for the indicated times and nuclear and cytoplasmic extracts were prepared. The experiments were performed in triplicate and the results are provided as mean values from three independent experiments.

### Isolation of human chondrocyte cytoplasmic and nuclear extracts

Isolation of cytoplasmic and nuclear extracts was performed as previously described in detail [33]. Briefly, primary human chondrocytes in monolayer cultures were trypsinized and washed twice in 1 ml ice-cold PBS. The cell pellet was resuspended in 400 µl hypotonic lysis buffer containing protease inhibitors and incubated on ice for 15 min. Some 12.5 µl of 10% NP-40 were added and the cell suspension vigorously mixed for 15 seconds. The extracts were centrifuged for 1.5 min. The supernatants (cytoplasmic extracts) were frozen at -70°C. Approximately 25 µl of ice-cold nuclear extraction buffer was added to the pellets and incubated for 30 min. Extracts were centrifuged and the supernatant (nuclear extracts) transferred to pre-chilled tubes for storage at -70°C.

### Western blot analysis and immunoblotting

Immunoblotting was performed as described in detail by Shakibaei *et al*. [34,35]. Cell proteins were extracted with lysis buffer (50 mM Tris/HCl, pH 7.2, 150 mM NaCl, l% (v/v) Triton X100, 1 mM sodium orthovanadate, 50 mM sodium pyrophosphate, 100 mM sodium fluoride, 0.01% (v/v) aprotinin, 4 µg/ml pepstatin A, 10 µg/ml leupeptin, 1 mM phenylmethylsulfonyl-fluoride (PMSF)) on ice for 30 min. For immunoblotting, the total proteins were separated by SDS-PAGE on a 10% SDS-polyacrylamide gel under reducing conditions. Subsequently, the proteins were transferred for 60 min at 120 V onto a nitrocellulose membrane (Schleicher & Schüll, Dassel, Germany) using a transblot electrophoresis apparatus (Mini Trans Blot™, Bio-Rad Laboratories, Richmond, CA, USA). The membranes were then blocked with a blocking buffer, containing 5% (w/v) skimmed milk powder in PBS/0.1% Tween 20, overnight at 4°C.

Immunoblotting with primary antibodies was performed for 1 h at room temperature (RT), followed by three washes in the blocking buffer. Subsequently, incubation with secondary antibody conjugated to alkaline phosphatase was performed for 30 min at RT. All antibodies were diluted with blocking buffer. After three washes in blocking buffer and two washes in 0.1 M Tris, pH 9.5, containing 0.05 M MgCl_2 _and 0.1 M NaCl, color development was performed using nitro blue tetrazolium and 5-bromo-4-chloro-3-indoyl-phosphate (p-toluidine salt) (Pierce, Rockford, IL, USA) as substrates for alkaline phosphatase.

### Co-immunoprecipitation of LPS and TLR4

Primary human chondrocyte cultures were treated either with LPS (100 ng/ml) or left untreated overnight. The medium and unbound LPS were then removed. The chondrocytes were washed three times and whole-cell extracts were prepared, immunoprecipitated [34] with an anti-LPS antibody, and precipitates were subjected to western blot analysis using an anti-TLR4 antibody.

### Immunofluorescence analysis of TLR4 and NF-κB

The effect of LPS on TLR4 and NF-κB translocation from the chondrocyte cytoplasm to the nucleus in response to NF-κB-activation by LPS was investigated by an immunocytochemical method, as previously described in detail [36]. Briefly, cells were seeded on glass plates and incubated for 24 h. The cells were rinsed three times and preincubated for 1 h with serum-starved medium, and then stimulated with 100 ng/ml LPS or BMS-345541 (5 mM) alone or prestimulated with BMS-345541 (5 mM) for 12 h before treating with LPS (100 ng/ml) for an additional 24 h in serum-starved medium. Glass plates were rinsed three times in PBS before methanol fixation and permeabilization of the cell and nuclear membranes for 1 h at ambient temperature (AT). Cells were overlaid with protease-free bovine serum albumin (BSA) for 10 min at AT, rinsed with PBS and incubated with primary antibodies (TLR4, phospho p65, 1:30 in PBS/BSA) in a humid chamber overnight at 4°C. They were gently washed several times with PBS/BSA before incubation with rhodamine red-conjugated secondary antibody (diluted 1:50 in PBS) for 1 h at AT and finally washed again three times with aqua dest. Counterstaining was performed with DAPI to visualize the cell nuclei. Samples were evaluated under a light microscope (Leica, Wetzlar, Germany) and photomicrographs were digitally stored.

### Immune complex kinase assay

To evaluate the effect of endotoxin on IKK activation, immune complex kinase assays were performed. The assay was performed as described in detail by Shakibaei *et al*. [37]. Briefly, the IKK complex was immunoprecipitated from whole cell lysates with antibodies against IKK-α and IKK-β and subsequently incubated with protein A/G-agarose beads (Pierce, Ulm, Germany). After a 2 h incubation, the beads were washed with lysis buffer and resuspended in a kinase assay solution containing 50 mM HEPES (pH 7.4), 20 mM MgCl2, 2 mM dithiothreitol, 10 mM unlabeled ATP and 2 mg substrate GSTIκBa (amino acid 1-54) and incubated at 30°C for 30 min. This was followed by boiling in SDS-PAGE sample buffer for 5 min. The proteins were transferred to a nitrocellulose membrane after SDS-polyacrylamide gel electrophoresis under reducing conditions as described above. Phosphorylation of GST-IκBα was assessed using a specific antibody against phosphospecific IκBα (Ser 32/36). To demonstrate the total amounts of IKK-α and IKK-β in each sample, whole-cell lysates were transferred to a nitrocellulose membrane after SDS-polyacrylamide gel electrophoresis under reducing conditions as described above.

### Transmission electron microscopy (TEM)

A detailed description of the culture technique used for transmission electron microscopy has been published [32,36]. After fixation and post fixation in 1% tannic acid (0.1 M phosphate buffer) and 1% OsO_4 _solution (0.1 M phosphate buffer), cartilage high-density cultures were rinsed and dehydrated in ascending alcohol series. They were embedded in Epon, cut on a Reichert Ultracut (Leica, Wetzlar, Germany) followed by contrasting with 2% uranyl acetate/lead citrate. For inspection a transmission electron microscope (EM 10 Zeiss, Institute of Pharmacology, Berlin, Germany) was used.

### Immunoelectron microscopy

A detailed description of the culture technique used for immunoelectron microscopy has been published [32,36]. High-density cultures were washed three times in PBS before fixation in 3% formaldehyde freshly prepared from paraformaldehyde plus 0.25% glutaraldehyde in PBS for 1 h. Then, the cultures were washed with PBS/1% BSA, dehydrated in ethanol and embedded in LR-white. Ultrathin sections were cut and treated with the following solutions: (1) 1% BSA at AT for 30 min; (2) testicular chondroitinase (5000 U/ml) for 5 min at AT to unmask epitopes; (3) PBS/1% BSA/0.5% Tween 20 2 × 5 min at AT; (4) primary antibodies (1:50 in PBS/1% BSA/0.5% Tween 20) overnight at 4°C; (5) PBS/BSA/Tween for 2 × 5 min at AT; (6) secondary antibodies conjugated with goat anti-rabbit immunoglobulin with 10 nm gold particles (1:50 for 30 min) at AT. (7) After rinsing for 2 × 5 min at AT, (8) contrasting was carried out with 1% tannic acid for 20 min at AT, with OsO_4 _for 10 min and with 2% uranyl acetate for 30 min. Finally, the sections were rinsed and examined under a transmission electron microscope (TEM 10 Zeiss, Institute of Pharmacology, Berlin, Germany).

### Pharmacological experiments with BMS-345541 or/and wortmannin

Primary human chondrocytes were grown in growth medium for 24 h. NF-κB or PI-3K inhibition experiments were carried out in serum-starved medium (0.5% FCS). Serum-starved chondrocytes were stimulated with LPS alone (100 ng/ml) or with wortmannin (20 nM), BMS-345541 (5 mM) or prestimulated with wortmannin (20 nM), BMS-345541 (5 mM) for 12 h before treating with LPS (10 to 1000 ng/ml) for an additional 24 h. LPS or inhibitors concentrations used in this study are the same as those in previous studies from our own laboratory and were calculated through dose-dependent experiments on human articular chondrocytes [19]. After these treatments, nuclear extracts were prepared and examined for NF-κB and/or PI3k/Akt as described above.

## Results

We examined the effect of endotoxin (LPS) on NF-κB and PI-3K activation pathways and NF-κB-regulated gene expression. The studies were performed on primary human chondrocytes, as these cells are one of the primary targets of LPS during inflammatory processes in rheumatic diseases such as RA.

### Presence of LPS (endotoxin) in the cartilage ECM in high-density culture in vitro

**Figure 1:**
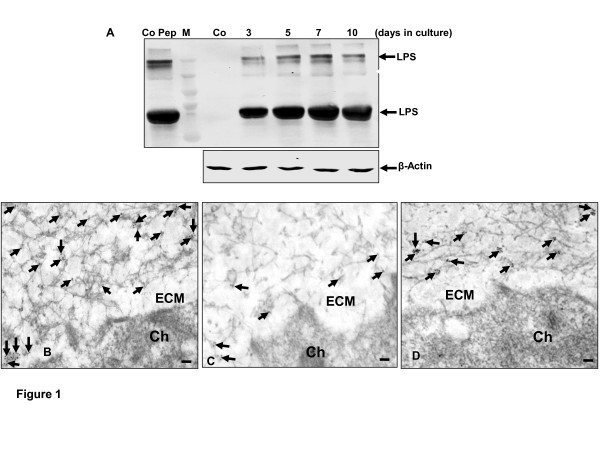
**Presence of LPS in the extracellular matrix in cartilage tissue**. **(A) **Primary human chondrocytes (1 × 10^6 ^cells) were cultured in high-density culture with lipopolysaccharides (LPS) (100 ng/ml) or without LPS (Co) for different times. Whole-cell extracts were prepared, and cell lysates were resolved by SDS-PAGE, electrotransferred to nitrocellulose membrane, and then probed for LPS expression by western blot analysis using antibodies to this protein. β-Actin served as an internal control. LPS control peptide (Co Pep) was used as a control. M = marker for molecular weights. **(B) **Primary human chondrocytes (Ch) (1 × 10^6 ^cells) were cultured for 7 days in high-density culture and then incubated with LPS (100 ng/ml) for 12 h before evaluation by immunoelectron microscopy. Secondary gold particle-labeled antibody was observed directly on the collagen bundles or clustered (arrows) together indicating the existence of LPS between the mesh of the collagen fibers in the extracellular matrix (ECM). **(C-D) **Primary human chondrocytes (Ch) (1 × 10^6 ^cells) were cultured for 7 days in high-density culture, and then first preincubated with anti-collagen type II (100 ng/ml) (C) or control rabbit IgG (100 µl/ml) (D) for 24 h and followed by incubation with LPS (100 ng/ml) for 12 h before evaluation with immunoelectron microscopy. Opposite to preincubated cultures with control rabbit IgG, only a very small amount of secondary gold particle-labeled antibodies was observed on the collagen bundles or clustered (arrows) in the extracellular matrix (ECM) in preincubated cultures with anti-collagen type II. Magnification x35,000; bar, 0.25 µm.

To visualize LPS accumulation in the cartilage matrix, we established high-density cultures and treated them with LPS (100 ng/ml) for 3, 5, 7 and 10 days. In untreated cultures of cartilage tissue, LPS was not detected (Figure 1A). In contrast to this, in high-density cultures treated with LPS, the presence of LPS was significantly increased in a time-dependent manner, as shown by immunoblotting assay (Figure 1A).

To visualize the presence, localization and the interaction with the ECM compound in cartilage tissue, we performed immunoelectron microscopy. After 7 days of solitary cultivation in high-density cultures, co-cultivation with LPS (100 ng/ml) for 12 h was performed (Figure 1B-C). High-density cultures show typical cartilaginous tissue with chondrocytes and appropriate matrix had developed. Labeling with LPS antibodies revealed the gold particles to be quite irregularly distributed in the matrix, formed clusters and were concentrated predominantly at collagen fibrils in the matrix (Figure 1B). To determine whether antibodies with the capacity to block certain binding epitopes on the collagen matrix, could have any effect on LPS accumulation on the collagen fibers, after 7 days of solitary cultivation in high-density cultures, preincubation with anti-collagen type II (100 ng/ml) or with 100 µl/ml control rabbit IgG for 24 h and then incubation with LPS (100 ng/ml) for 12 h were performed. The collagen type II antibodies were found to significantly reduce the binding of LPS to collagen type II compared with LPS treatment alone, which indicates the involvement of LPS binding to certain epitopes on the collagen fibers (Figure 1C). In contrast to this, treatment with control rabbit IgG alone showed abundant accumulation of LPS on the collagen fibers in the matrix (1D).

### BMS-345541 or/and wortmannin suppress LPS-induced degenerative features and apoptosis in chondrocytes and promote differentiation of LPS-treated chondrocytes in high-density culture

**Figure 2:**
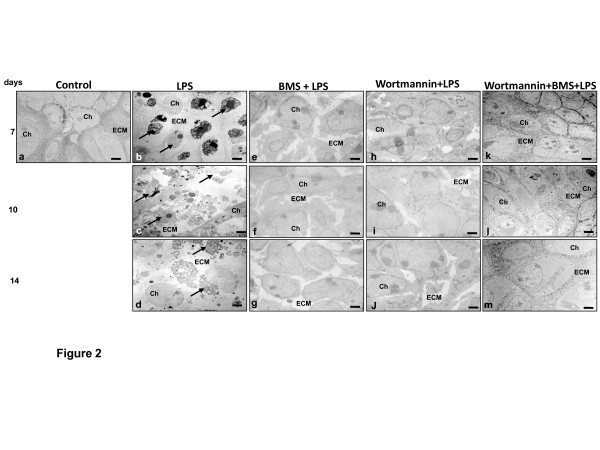
**LPS induced cellular and matrix degradation and apoptosis in cartilage tissue**. Primary human chondrocytes were either left untreated **(a) **or were treated with 100 ng/ml lipopolysaccharides (LPS) **(b-d)**, pretreated with BMS-345541 (5 mM) **(e-g)**, wortmannin (20 nM) **(h-j) **followed by LPS treatment, or pretreated in combination with BMS-345541 and wortmannin (5 mM and 20 nM) **(k-m) **for 12 h and then stimulated with LPS for another 24 h. The cells were transferred to high-density culture for 14 days. Ultrastructural morphology was evaluated by electron microscopy. Control cultures of chondrocytes showed well-developed chondrocytes (Ch) embedded in a well-developed extracellular matrix (ECM) (a). Treatment with LPS resulted in matrix breakdown and cell lysis and apoptosis (arrows) (b-d). Pretreatment with BMS-345541 alone (e-g), with wortmannin alone (h-j) or in combination with BMS and wortmannin (k-m) resulted in a marked improvement of chondrocyte phenotype and the formation of cartilage nodules. The formation of a dense extracellular matrix (ECM) surrounding well-developed chondrocytes (Ch) was observed. Magnification x5000; bar, 1 µm.

Our group has previously reported that high-density culture promotes chondrocyte differentiation since it supports cell-cell interactions necessary for adequate matrix formation [32,38]. High-density cultures were prepared from chondrocytes of monolayer passages 3 and cultivated for 7 days and prepared for transmission electron microscopy. After 7 days in high-density cultures, primary human chondrocytes showed well-developed cartilage nodules with viable cells and well-developed and organized cell organelles, such as rER, mitochondria, glycogen granules, free cytoplasmic ribosomes, Golgi apparatus. The cells were embedded in an extensive fine fibrillar matrix tightly attached to the cytoplasmic membrane (Figure 2, panel a).

LPS treatment (100 ng/ml) resulted in cell lysis, degenerative and apoptotic features including formation of dense materials in nuclei, formation of blebs at the cell surface, formation of apoptotic bodies and degeneration of ECM structure (Figure 2, panel b). After longer incubation periods (10 to 14 days), more severe features of cellular degeneration such as cell lysis, extensive matrix breakdown and some characteristic features of apoptotic cell death (Figure 2, panels c and d) were seen.

We tested whether BMS-345541 or/and wortmannin can modulate LPS-induced lysis and apoptosis of chondrocytes in high-density culture. In contrast to LPS treatment alone, pretreatment with either BMS-345541 (5 mM, specific inhibitor of the IKKβ) (Figure 2, panels e-g) or wortmannin (20 nM, a blocker of PI-3K signaling) (Figure 2, panels h-j) alone or in combination BMS-345541 and wortmannin (5 mM and 20 nM) (Figure 2, panels k-m) significantly reduced the cytotoxic and apoptotic effects of LPS. This demonstrates that BMS-345541 and wortmannin inhibit the cytotoxic and apoptotic effects induced by LPS in chondrocytes. Interestingly, co-treatment of the chondrocytes with both blockers inhibited these effects more than each agent by itself. Taken together, these findings suggest that the NF-κB and PI-3K signaling pathways play (at least in part) an important role in the destructive effects of LPS in chondrocytes.

### *BMS-345541 or/and wortmannin suppress *LPS-induced NF-κB-dependent proinflammatory, proapoptotic and matrix-degrading gene products in chondrocytes

**Figure 3:**
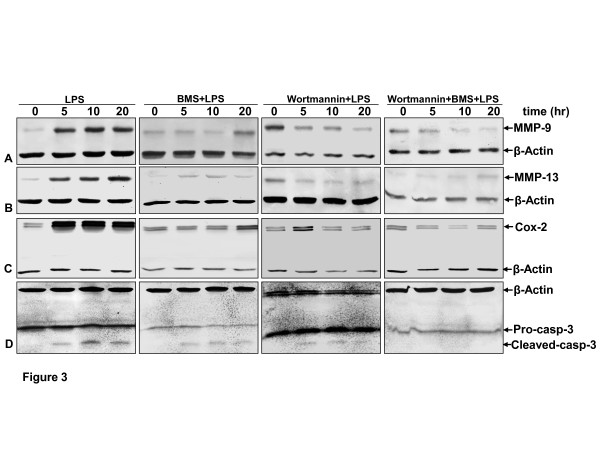
**Effects of BMS-345541 or/and wortmannin on the LPS-induced proinflammatory and apoptotic signaling in human chondrocytes**. Primary human chondrocytes in monolayer cultures were either left untreated or stimulated with 100 ng/ml lipopolysaccharides (LPS), prestimulated with 5 mM BMS-345541, with 20 nM wortmannin or a combination of both inhibitors, BMS-345541 and wortmannin (5 mM and 20 nM) for 12 h and then treated with 100 ng/ml LPS for the indicated times. Whole-cell extracts were fractionated (500 ng protein per lane) on SDS-PAGE and examined by western blot analysis using anti-matrix metalloproteinase (MMP)-9 **(A)**, -MMP-13 (**B)**, -cyclooxygenase-2 (Cox-2) **(C) **and anti-cleaved caspase-3 **(D)**. The results shown are representative of three independent experiments. Housekeeping protein β-actin served as a loading control.

Further, we examined whether BMS-345541 or/and wortmannin can modulate the activation of LPS-induced NF-κB-regulated gene products involved in the inflammation and degradation processes in cartilage tissue. It has been previously reported that inflammatory agents activate COX-2, MMP-9, MMP-13 and caspase-3 through the NF-κB signaling pathway [29]. COX-2 is an enzyme that catalyzes the production of prostaglandin E2 (PGE2) from arachidonic acid, which is an important inflammatory mediator that has been linked to the pathogenesis of RA and OA [39]. MMPs play an important role in the pathogenesis of RA and OA by promoting angiogenesis in the synovial joint and facilitating infiltration of inflammatory cells in the synovial joint by virtue of its property to degrade extracellular matrix [40]. Hence, primary human chondrocytes cultured with or without pretreatment with BMS-345541 or/and wortmannin were examined for LPS-induced gene products by western blot analysis using specific antibodies (Figure 3). Treatment with LPS alone (100 ng/ml) induced the expression of COX-2, MMP-9, MMP-13, and cleavage of caspase-3 in a time-dependent manner (Figure 3A-D, panel 1). In contrast to this, pretreatment with BMS-345541 or wortmannin significantly inhibited the expression of the mentioned genes and cleavage of caspase-3 (Figure 3A-D, panel 2, 3). Combinational pretreatment of the inhibitors was effective in inhibition of these proinflammatory proteins in the same manner in chondrocytes (Figure 3A-D, panel 4).

### Suppressive effect of BMS-345541 or/and wortmannin on LPS-induced phosphorylation and translocation of NFκB-p65 in nuclear extracts of chondrocytes in a dose- and time-dependent manner

**Figure 4:**
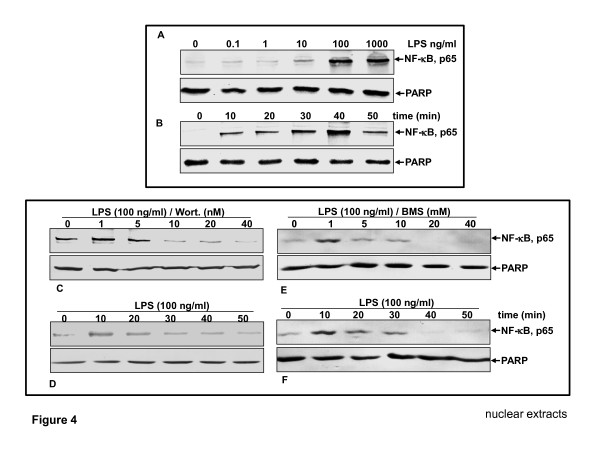
**LPS induced the phosphorylation and translocation of p65 and the IKK inhibitor BMS-345541 or PI-3K inhibitor wortmannin suppressed this in a time- and dose-dependent manner**. **(A-B) **Primary human chondrocytes in monolayer culture were either left untreated (controls), treated with lipopolysaccharides (LPS) (0, 0.1, 1, 10, 100 and 1000 ng/ml) for 30 min (A) or stimulated with 100 ng/ml LPS for the indicated times (B). Nuclear extracts were prepared and assayed for nuclear factor-κB (NF-κB) (p65) activation by western blot analysis as described in Materials and methods. **(C-F) **Primary human chondrocytes in monolayer cultures were either left alone or pretreated with BMS-345541 or with wortmannin for the indicated concentrations followed by treatment with 100 ng/ml LPS for 30 min (C and E), or prestimulated with 5 mM BMS-345541 or with 20 nM wortmannin for 12 h and co-treated with 100 ng/ml LPS for the indicated times (D and F). Nuclear extracts were prepared and assayed for NF-κB (p65) activation by western blot analysis as described in Materials and methods. Synthesis of poly(ADP-ribose) polymerase (PARP) remained unaffected in nuclear extracts. IKK, IκB kinase; PI-3K, phosphatidylinositol 3-kinase.

First, the optimum dose and time of exposure to LPS required to induce NF-κB activation has been determined (data not shown). To evaluate whether LPS induces activation of NF-κB, nuclear protein extracts of serum-starved human chondrocytes were probed for the phosphorylated p65 NF-κB subunit after treatment with the indicated concentrations of LPS for 30 min (Figure 4A). As indicated by western blotting analysis, LPS induced NF-κB activation in a dose-dependent manner (Figure 4A). We next investigated whether activation of NF-κB by LPS is also time dependent. For this, primary human chondrocytes were incubated with 100 ng/ml LPS for the indicated times. Western blotting results showed that activation of NF-κB by LPS was also found to be time-dependent (Figure 4B). Taken together, these findings indicate that the activation and translocation of NF-κB by LPS is dose- as well as time-dependent.

We next focused on the mechanistic relationship between LPS effects and NF-κB signaling pathways. BMS-345541 is a potent and specific IKK inhibitor and can effectively inhibit NF-κB activation induced by diverse stimuli [41]. Wortmannin is a specific inhibitor of PI-3K signaling [24]. Therefore, we treated primary chondrocytes with BMS-345541 or/and wortmannin to determine whether the effects of LPS on chondrocytes are also associated with an altered activation status of the IKK-NF-κB and PI-3K pathways and whether it suppressed the destructive effects of LPS. Serum-starved chondrocytes were pretreated with BMS-345541 or/and wortmannin and then co-treated with LPS. As shown in Figure 4, pretreatment with BMS-345541 or/and wortmannin inhibited the LPS-induced phosphorylation and translocation of p65 to the nucleus in a time- and dose-dependent manner (Figure 4C-F).

### BMS-345541 inhibits LPS-induced nuclear translocation of p65 as revealed by immunofluorescence microscopy

**Figure 5:**
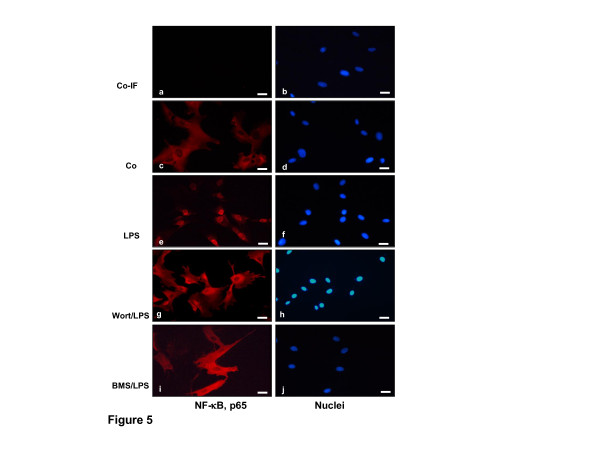
**BMS-345541 or wortmannin inhibited LPS-induced nuclear translocation of phospho p65 in chondrocytes as demonstrated by immunofluorescence microscopy**. Primary human chondrocyte cultures either served as controls (**a-b**: without primary antibody; **c-d**: with primary antibody) or were treated with lipopolysaccharides (LPS) alone **(e-f) **or were pre-treated with wortmannin (20 nM) **(g-h) **or with BMS-345541 (5 mM) **(i-j) **for 12 h before co-treatment with LPS (100 ng/ml) for 24 h before immunolabeling with phospho p65 antibodies and rhodamine-coupled secondary antibodies and counterstained with DAPI to visualize cell nuclei. Images shown are representative of three independent experiments. Magnification x400; bar, 30 nm.

Based on the western blotting results (Figure 4) and to confirm them, we performed immunocytochemical analysis. Primary human chondrocytes either served as controls (not treated: Co-IF, without primary antibody, Figure 5, a-b; not treated: basal co, with primary antibody, Figure 5, c-d) or were stimulated with BMS-345541 alone (data not shown), with wortmannin alone (data not shown) or with 100 ng/ml LPS alone for 12 h (Figure 5, e-f) or were co-treated with wortmannin (20 nM) (Figure 5, g-h) or with BMS-345541 (5 mM) (Figure 5, i-j) for 12 h before treating with LPS (100 ng/ml) for 24 h before indirect immunolabeling with anti-phospho p65 antibodies and rhodamine-coupled secondary antibodies. Counterstaining was performed with DAPI to visualize the cell nuclei. Immunofluoroscence microscopy showed clear and intensive cytoplasmic and nuclear staining for phospho p65 in primary human chondrocytes treated with LPS (Figure 5, e-f). In contrast to this, co-treatment of chondrocytes with LPS and BMS-345541 or wortmannin resulted in decreased nuclear staining of activated phospho p65 and indicated a decrease in activation of NF-κB (Figure 5, g-j). Control chondrocytes and chondrocytes treated with BMS-345541 or wortmannin alone (data not shown) showed only cytoplasmic labeling of phospho p65 (Figure 5, c-d). These immunomorphological findings were consistent with the NF-κB inhibition observed by western blotting. Images shown are representative of three independent experiments.

### BMS-345541 or wortmannin inhibits LPS-induced-IκBa degradation and phosphorylation in chondrocytes

**Figure 6:**
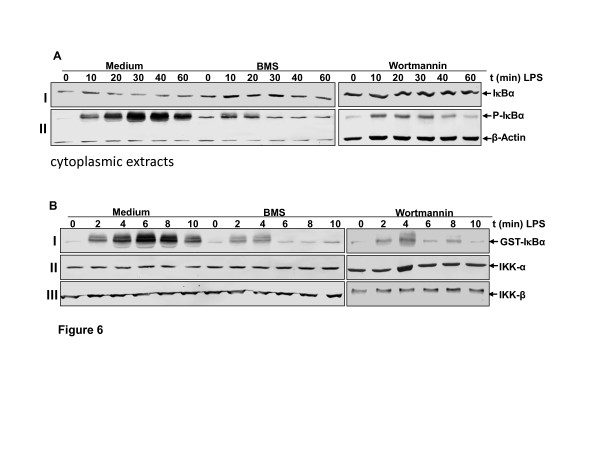
**Effects of BMS-345541 and wortmannin on LPS signalling in chondrocytes**. **(A) Effects of BMS-345541 or wortmannin on LPS-induced IκB-α phosphorylation and degradation in chondrocytes in monolayer cultures**. Primary human chondrocytes in monolayer culture were either stimulated with 100 ng/ml lipopolysaccharides (LPS) or prestimulated with 5 mM BMS-345541 or with 20 nM wortmannin for 12 h followed by LPS treatment (100 ng/ml) for the indicated times. Cytoplasmic extracts were prepared, fractionated (500 ng protein per lane) on 10% SDS-PAGE and electrotransferred onto nitrocellulose membranes. Western blot analysis was performed with anti-phospho-specific-IκB-α (II), anti-IκB-α (I) and anti-β-actin (II, control). The results shown are representative of three independent experiments. **(B) Effects of BMS-345541 or wortmannin on LPS-induced IKK activation in chondrocytes in monolayer cultures**. Primary human chondrocytes in monolayer culture were either stimulated with 100 ng/ml LPS or prestimulated with 5 mM BMS-345541 or with 20 nM wortmannin for 12 h followed by treatment with 100 ng/ml LPS for the indicated times. Whole-cell extracts were immunoprecipitated with an antibody against IkB kinase (IKK) and then analyzed by an immune complex kinase assay as described in Materials and methods. To examine the effect of LPS, BMS-345541 or wortmannin on the level of activation of IKK proteins, whole-cell extracts were fractionated (500 ng protein per lane) on SDS-PAGE and examined by western blot analysis using anti-phospho-specific-IκB-α and anti-IκB-β (I). LPS, BMS-345541 or wortmannin had no direct effect on the expression of IKK-α or IKK-β proteins (II, III). The results shown are representative of three independent experiments.

BMS-345541 or wortmannin inhibited LPS-induced activation and translocation of NF-κB to the chondrocyte nucleus. Therefore, we evaluated the upstream mechanisms of NF-κB activation by LPS in chondrocytes. It has been reported that the phosphorylation and degradation of IκBα, the natural blocker of NF-κB, is a prerequisite for the activation of NF-κB [42]. To examine whether inhibition of LPS-induced NF-κB activation occurs through inhibition of IκBα degradation, we treated cells with BMS-345541 or wortmannin, followed by LPS stimulation and probed them for IκBα activation in the cytoplasm by western blot analysis. LPS induced IκBα degradation in control cells as early as 20 min (Figure 6A, I) but in co-treated cultures the degradation of IκBα was not evident (Figure 6A, I). These results indicate that LPS induces IκBα degradation by acting at an upstream step to NF-κB activation. Furthermore, LPS-induced IκBα phosphorylation was almost completely blocked by BMS-345541 or wortmannin (Figure 6A, II). These results indicate that BMS-345541 or wortmannin inhibits both LPS-induced IκBα degradation and phosphorylation. Data shown are representative of three independent experiments. Taken together, these results suggest that BMS-345541 or wortmannin block LPS-induced IκBα degradation.

### Effect of BMS-345541 or wortmannin on LPS-induced activation of IKK in chondrocytes in monolayer cultures

IKK is required for phosphorylation of IκBα [42]. To determine whether BMS-345541, the specific inhibitor of IKKβ [41] or wortmannin a specific inhibitor of PI-3K signaling [24] can inhibit the NF-κB pathway, we tested the effect of BMS-345541 or wortmannin on LPS-induced IKK activation, which is required for LPS-induced phosphorylation of IκBα. Immune complex kinase assays showed that LPS induced the activation of IKK in a time-dependent manner. In contrast to this, pretreatment of chondrocytes with BMS-345541 or wortmannin followed by stimulation with LPS resulted in an inhibition of LPS-induced effects on the activation of IKK (Figure 6B, I). LPS, BMS-345541 or wortmannin had no direct effect on the expression of IKK-α or IKK-β proteins (Figure 6B, II, III).

### The effect of wortmannin (PI-3K inhibitor) on LPS-induced phosphorylation of PI-3K/Akt in chondrocytes

**Figure 7:**
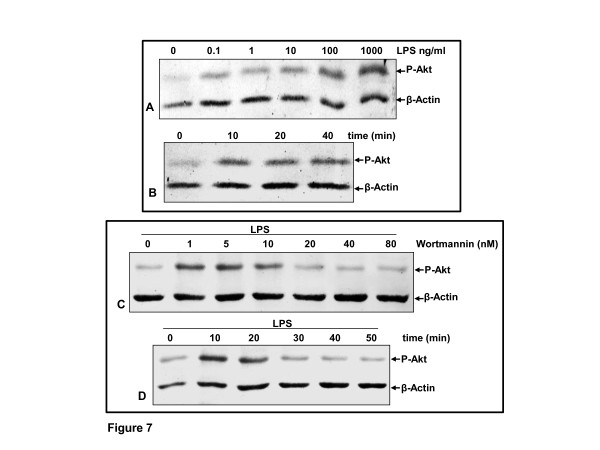
**LPS induced PI-3K/Akt activation and PI-3K inhibitor (wortmannin) suppressed this in a time- and dose-dependent manner**. **(A-B) **Primary human chondrocytes in monolayer culture were either left untreated (controls), treated with lipopolysaccharides (LPS) (0, 0.1, 1, 10, 100 and 1000 ng/ml) for 50 min or stimulated with 100 ng/ml LPS for the indicated times. Nuclear extracts were prepared and assayed for Akt activation by western blotting as described in Materials and methods. **(C-D) **Primary human chondrocytes in monolayer cultures were either left untreated or were prestimulated with wortmannin for the indicated concentrations and treated with 100 ng/ml LPS for 50 min, or prestimulated with 20 nM wortmannin for 12 h followed by co-treatment with 100 ng/ml LPS for the indicated times. Nuclear extracts were prepared and assayed for Akt activation by western blot analysis as described in Materials and methods. Housekeeping protein β-actin served as a loading control and remained unaffected. PI-3K, phosphatidylinositol 3-kinase.

It has been reported that the phosphatidylinositol 3-kinase phosphorylation of endogenous Akt pathway is required for the activation of NF-κB, as an upstream protein kinase, and wortmannin is a specific inhibitor of PI-3K signaling [24]. Next, therefore, we determined whether LPS induces phosphorylation of PI-3K/Akt in chondrocytes. As indicated by western blotting using an antibody specific for phosphorylated Akt, LPS induced Akt phosphorylation greatly in a dose-dependent manner (Figure 7A). Further, we examined whether activation of Akt by LPS is also time dependent. Primary human chondrocytes were incubated with 100 ng/ml LPS for the indicated times. Western blotting showed that activation of Akt by LPS was also found to be time dependent (Figure 7B).

To examine the role of the PI-3K/Akt signaling pathway in regulating LPS-mediated NF-κB activation, the level of Akt phosphorylation protein was analyzed using selective kinase inhibitors. Chondrocytes were pretreated with inhibitors of PI-3K (wortmannin), respectively for 1 h, and then co-treated with 100 ng/ml LPS for 1 h. As shown in Figure 7C-D, the activation of Akt (upstream protein kinase B) in chondrocytes was significantly reduced by preincubation with wortmannin in a time- and dose-dependent manner. These results further suggest that LPS-induced NF-κB activation in chondrocytes, at least in part, is regulated through the PI-3K/Akt signaling pathway.

### LPS induces the expression of and physically binds to TLR4 in human chondrocytes

To better understand the mechanism whereby LPS induces degradation of cartilage and ECM, activation of p65 and PI-3K and degradation of IκBα, we examined the activation and binding of TLR4 with LPS on human chondrocytes following LPS treatment. Indeed, it has been reported that LPS is the primary ligand of the Toll-like receptor 4 (TLR4) [18].

**Figure 8:**
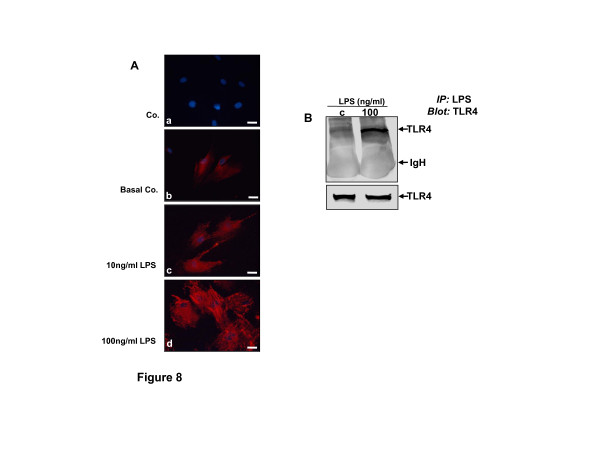
**LPS induced TLR4 expression and associated with TLR4 in human chondrocytes**. **(A) **Primary human chondrocytes were either left untreated (**b**, basal Co) or were treated with 10 ng/ml **(c) **or 100 ng/ml lipopolysaccharides (LPS) **(d) **for 12 h, then were immunostained with anti-Toll-like receptor 4 (TLR4) (red) and counterstained with DAPI (blue). Control, in which primary antibodies were replaced with control rabbit IgG **(a)**. Results are representative of three experiments using human chondrocytes. Magnification x400; bar, 30 nm. **(B) **Lysates prepared from human chondrocytes exposed to LPS (100 ng/ml) or untreated (c) (negative control) were incubated with anti-LPS. Immunoprecipitates were analyzed by western blotting with anti-TLR4. Presence of band of the correct size (approximately 100 kDa) corresponding to chondrocytes treated with LPS confirms the binding of LPS to TLR4. The original samples were probed with an antibody to anti-TLR4. Data shown are representative of three independent experiments. IgH: immunglobulin heavy chain.

TLR4 expression is low in normal untreated human chondrocytes (Figure 8A). We asked whether TLR4 expression is increased and characterized a subset of LPS in chondrocytes. To address this question, we treated the chondrocytes in monolayer cultures with different concentration of LPS (10 or 100 ng/ml) for 12 h or left them untreated and examined them with immunofluorescent staining for TLR4. As shown in Figure 8A: a-d, the expression of TLR4 is clearly increased by LPS and this was dose dependent. These data suggest that LPS induces TLR4 expression, as its receptor in inflamed chondrocytes. Therefore, we further examined the effect of LPS on TLR4 and LPS-induced LRP-TLR4 association by a co-immunoprecipitation assay. Primary human chondrocytes were treated with 100 ng/ml LPS for 4 h or left untreated (c). After 4 h of incubation, the media were replaced with the regular medium, lysed and then co-immunoprecipitation assays were performed. After immunoprecipitation with anti-LPS antibodies, the samples were probed by immunoblotting with anti-TLR4. The results indicate that LPS was co-immunoprecipitated by anti-TLR4 antiserum but not by control cultures (Figure 8B). As a control, the immunoprecipitation by a control IgG did not result in precipitation of TLR4 (not shown). Taken together, these results indicate that LPS-TLR4 complex formation is one of the major pathways, which activates the NF-κB and PI-3K pathways in chondrocytes.

## Discussion

The data presented in this manuscript provides evidence to support the idea that endotoxins distributed in the cartilage matrix form clusters and concentrate predominantly at the frayed end of collagen fibrils in the matrix (procollagen-endotoxin complexes, PEC), suggesting that collagens and the ECM may act as a reservoir for endotoxins.

We have made the following novel observations: (I) as demonstrated by immunoelectron microscopy and western blot analysis, we showed the presence of LPS in cartilage matrix bound to the collagen fibrils and anti-collagen type II significantly reduced this interaction. (II) LPS-induced massive cartilage matrix break down and chondrocytes apoptosis is blocked in part by BMS-345541 (IKK-inhibitor), and was completely inhibited by the combinational pretreatment of BMS-345541 and wortmannin (a specific inhibitor of the PI-3K/Akt pathway), suggesting that NF-κB and PI-3K pathways are involved in LPS-induced cartilage degradation. (III) Wortmannin potentiates the anti-inflammatory and anti-apoptotic effects of BMS-345541 on LPS-stimulated chondrocytes, and this correlates with downregulation of NF-κB-specific gene products that are known to mediate inflammation, degradation and apoptosis of chondrocytes in OA and RA. (IV) LPS induced activation and translocation of p65 from the cytoplasm to the nucleus in a dose- and time-dependent manner and these effects were inhibited by BMS-345541 or/and wortmannin. (V) Suppression of NF-κB activation by BMS-345541 or/and wortmannin is due to inhibition of LPS-induced IKK activation, which led to inhibition of IκBα phosphorylation and degradation and suppression of p65 phosphorylation and its translocation to the nucleus. (VI) LPS induced the PI-3K/Akt pathway and this was inhibited by wortmannin. (VII) Finally, LPS stimulated TLR4 and, associated with TLR4, initiated NF-κB and PI-3K activation pathways.

A number of studies have reported that the inhabitants of buildings with dampness through, for example, water damage have an increased risk of RA [19-21]. A connection between microbial infestation of buildings after water damage and RA manifestation in inhabitants has been observed, where symptoms of RA decreased in patients after removing damp walls [19]. Previous studies from our group reported that in primary isolated chondrocytes, bacterial toxins taken from damp walls in buildings with water damage dose-dependently increased MMPs production and suppressed collagen type II production *in vitro *[19]. Therefore, we assumed that very fine particles, or LPS, are inhaled by the inhabitants and are partly transported to the joints/cartilage through circulation.

**Figure 9:**
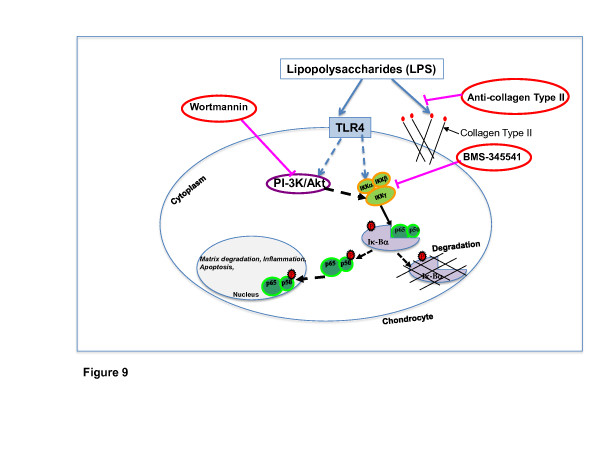
**Inhibitory effects of BMS-345541 or/and wortmannin on LPS-induced NF-κB/PI-3K and apoptosis in primary human chondrocytes *in vitro***. Lipopolysaccharides (LPS)-induced disruption of cartilage may be induced through LPS complex formation with collagen II fibrils and through LPS/Toll-like receptor 4 (TLR4) association. Anti-collagen type II significantly reduced these procollagen-endotoxin complexes. Functional association with TLR4 leads to activation of intracellular downstream signaling pathway NF-κB, nuclear factor-κB (NF-κB)/PI-3K, phosphatidylinositol 3-kinase (PI-3K) inducing upregulation of matrix-degrading enzymes, inflammation and apoptosis. Culturing with specific inhibitors wortmannin (for PI-3K/AKT) and BMS-345541 (for IκB kinases (IKKs)) suppresses LPS-induced inflammatory response indicating the potential for new medical approaches.

We hypothesized that LPS may interfere directly with the mechanisms for the synthesis and assembly of collagen fibers (Figure 9). Therefore, to perform new experiments with a well-defined compound, we chose LPS from *E.coli*. If LPS from *E.coli *has the same effects as the environmental LPS, it seems reasonable that the model we have developed will be valid for studying the effects of environmental noxa and systemic mediators of chronic bacterial inflammation for example peridontitis. In fact, binding of LPS to collagen type II can significantly be reduced by antibodies against collagen type II. This is compelling evidence for involvement of LPS binding to certain epitopes on the collagen fibers, suggesting the potential role of collagens as a reservoir for endotoxins. It is most likely that this binding between LPS and collagen type II influences cellular behavior (differentiation, cell shape, secretion products) and intracellular signaling. However, the molecular mechanism of LPS-collagen binding and thus control of ECM synthesis is at present only poorly understood. Interestingly, it has been already hypothesized that in obese patients with metabolic endotoxemia, that is high blood levels of LPS, caused by an impaired gastric mucosa may strongly contribute to the formation of OA [43].

The procollagens contain telopeptides at the C- and N-terminal ends, which have to be cleaved by specific catabolic enzymes (N- and C-proteinase) to render the mature tropocollagen molecule, which is then released from the cell [44]. The procollagen have frayed ends, which have a remarkable similarity to the ends of some Toll-like receptors (TLRs, specifically TLR4) [45], which are localized in the cell membrane of immune cells, especially macrophages [46]. We previously hypothesized that bacterial structures such as endotoxins bind to the end of procollagen in the cartilage ECM [19]. Thus, a stable procollagen-endotoxin complex (PEC) may form and through that collagen synthesis in chondrocytes may be disrupted.

We found that, BMS-345541, an inhibitor of NF-κB activation, blocked a part of the LPS-induced degradation of ECM and apoptosis, but this was completely inhibited by the combination of BMS-345541 and wortmannin, suggesting that NF-κB and PI-3K pathways are involved in LPS-induced cartilage degradation. We have also shown that LPS stimulates the expression of several proteins that are regulated by NF-κB, including proapoptotic protein caspase-3, the matrix-degrading MMPs, as well as the inflammatory enzyme COX-2 and this was blocked by BMS-345541 or/and wortmannin. Western blot analysis showed that BMS-345541 downregulated the activation of NF-κB by the inhibition of IκBα and IKK, suggesting the involvement of NF-κB in regulation of LPS-induced proapoptotic and degradative pathways in cartilage. These results support previous reports that have shown that LPS induces activation of NF-kB and downstream activities in normal or osteoarthritic mammalian chondrocytes [47-54]. In contrast to these studies, in this paper, we are showing for the first time that LPS stimulate the PI-3K/Akt signaling pathway in chondrocytes, which was inhibited by wortmannin, a specific inhibitor of the PI-3K/Akt pathway. This is also consistent with studies that have shown that NF-κB activation requires the PI-3K/Akt signaling pathway [55,56]. These findings explain, at least in part, the inflammatory and apoptotic effects of LPS in chondrocytes.

It has been reported that kinase Akt (protein kinase B) functions upstream of IKK [57]. Furthermore, previous studies have shown the inhibition of NF-κB to the DNA binding through the blocking of IκBα phosphorylation by the PI-3K/Akt pathway in various cell types [24,58-60]. However, downregulation of upstream signaling proteins, such as PI-3K/Akt by wortmannin, may be involved in LPS-mediated activation of NF-κB in chondrocytes.

We showed that LPS stimulated NF-κB/PI-3K pathways and these were suppressed by specific NF-κB/PI-3K inhibitors. Therefore, we approached to investigate whether LPS signaling acts through TLR4 (the primary receptor for LPS), in human chondrocytes. Indeed, we could demonstrate that LPS induced TLR4 expression on the surface of chondrocytes in a dose-dependent manner, which is consistent with a previous report [16,18]. Interestingly, immunoprecipitation assay and western blotting demonstrated functional and physical interactions between LPS and TLR4 in chondrocytes, suggesting that this complex initiates NF-κB and PI-3K activation pathways. Similar to our findings, recent studies in adipocytes have reported that LPS actively bind to adipocyte-expressed TLR4 inducing inflammation signaling in adipocytes [61,62].

## Conclusions

Our results suggest that LPS physically interact with collagen type II in the extracellular matrix and anti-collagen type II significantly reduced this interaction. Further, our study demonstrates, for the first time, that the blockade of LPS-induced activation of NF-κB and PI-3K pathways by specific inhibitors (BMS-345541 or wortmannin) explains the observed effects of LPS/TLR4 association on downstream proinflammatory responses, including the inhibition of cartilage ECM breakdown, inflammation and apoptosis in chondrocytes (Figure 9).

## Abbreviations

Akt, protein kinase B; AT, ambient temperature; COX-2, cyclooxygenase-2; DMEM, Dulbecco's modified Eagle's medium; ECM, extracellular matrix; FCS, fetal calf serum; IgH, immunglobulin heavy chain; IKK, IκB kinase; IL, interleukin; LPS, lipopolysaccharides; MAPK, mitogen-activated protein kinase; MMP, matrix metalloproteinase; NF-κB, nuclear factor-κB; NF, nuclear factor; OA, osteoarthritis; PARP, poly(ADP-Ribose) polymerase; PBS, phosphate-buffered saline; PEC, procollagen-endotoxin complex; PI-3K, phosphatidylinositol 3-kinase; PMSF, phenylmethylsulfonyl-fluoride; RA, rheumatoid arthritis; RT, room temperature; SDS-PAGE, sodium dodecyl sulfate-polyacrylamide gel electrophoresis; TLR4, Toll-like receptor 4; TNF, tumor necrosis factor.

## Competing interests

The authors declare that they have no competing interests.

## Authors' contributions

CB, CL and MS carried out all of the experiments and drafted the manuscript. WL and MS were responsible for the experiment design and data analysis. CB, AM and MS participated in histological and immunohistochemical analyses. WL, AM and MS participated in the evaluation of each experiment. WL and AM revised the paper and provided technical support and final edition of the paper. All authors read and approved the final manuscript.
